# Uncertainty in experts’ judgments exposes the vulnerability of research reporting anecdotes on animals’ cognitive abilities

**DOI:** 10.1038/s41598-021-95384-x

**Published:** 2021-08-10

**Authors:** Krisztina Sándor, Balázs Könnyű, Ádám Miklósi

**Affiliations:** 1 Behavioural Ecology Research Group, Center for Natural Sciences, University of Pannonia, Veszprém, Hungary; 2 MTA-ELTE Comparative Ethology Research Group, Budapest, Hungary; 3 Department of Plant Systematics, Ecology and Theoretical Biology, Eötvös Loránd University, Budapest, Hungary; 4 Institute of Evolution, Eötvös Loránd Research Network, Budapest, Hungary; 5 Department of Ethology, Eötvös Loránd University, Budapest, Hungary

**Keywords:** Biological techniques, Psychology

## Abstract

Expertise in science, particularly in animal behaviour, may provide people with the capacity to provide better judgments in contrast to lay people. Here we explore whether experts provide a more objective, accurate and coherent evaluation of a recently reported anecdote on Atlantic puffin (*Fratercula arctica*) “tool use” (recorded on video) which was published in a major scientific journal but was received with some scepticism. We relied on citizen science and developed a questionnaire to measure whether experts in ethology and ornithology and lay people agree or disagree on (1) the description of the actions that they observe (the bird takes a stick in its beak), (2) the possible goal of the action (nest-building or grooming) and (3) the intentional component of the action (the bird took the stick into its beak in order to scratch itself). We hypothesised that contrary to the lay people, experts are more critical evaluators that is they are more inclined to report alternative actions, like nest building, or are less likely to attributing goal-directedness to the action in the absence of evidence. In contrast, lay people may be more prone to anthropomorphise utilising a teleological and intentional stance. Alternatively, all three groups of subjects may rely on anthropomorphism at similar levels and prior expertise does not play a significant role. We found that no major differences among the evaluators. At the group levels, respondents were relatively uncertain with regard to the action of the bird seen on the video but they showed some individual consistency with regard to the description of the action. Thus, we conclude that paradoxically, with regard to the task our experts are typically not experts in the strict sense of the definition, and suggest that anecdotal reports should not be used to argue about mental processes.

## Introduction

Cognitive psychology and cognitive ethology rely on behaviour as a proxy for making hypotheses about mental functioning^1,2^. Since the early beginning of comparative psychology^3^ there has been a strong tradition to study animal behaviour under controlled conditions in the laboratory. While this approach is more in line with the expectations of an objective scientific inquiry, these experimental methods were often criticised for limiting external validity and generalisability with regard to the richness and complexity of animal behaviour in nature^4,5^.

Thus, following the footsteps of early ethologists, many scientists choose the ‘hard way’ and designed experiments under natural or semi-natural conditions (e.g.^6,7^) where controlling for both external (environmental) and internal (subject-specific) variables is much constrained but the face and construct validity of the experiment is enhanced.

However, an even older tradition of animal behaviour science is also still alive. Reporting observations on unique events involving single or few individuals are also published. For example, Appleby et al.^8^ described in detail the case of a lactating dingo (*Canis dingo*) carrying around the body of her dead puppy for many days. Such instances are usually referred to as anecdotes which are defined by common sense as reports on a rare behaviour or event that has been observed either once or few times (^9^; see also^10^).

Anecdotal reports may have some significant role in exposing interesting aspects of the animals’ (species) behaviour but the main problem is that these accidental observations are often used as a basis for arguments on mental mechanisms. For example, a recently published article reported “*Evidence of tool use in a seabird*”^11^ was based on a single accidental observation caught on video. The 11 s long footage shows an Atlantic puffin (*Fratercula arctica*) that puts a stick in its beak that gets into contact with the feathers on its belly. The authors claimed that this event was a case for ‘scratching’, revealing the capacity for ‘tool use’ in this species. Unfortunately, this report has ignored many important steps of scientific analysis that could have provided a more critical interpretation of the video recorded action^10^. Not surprisingly, the publication of the article was soon followed by a series of critical commentaries (^10,12–14^ but see^15^).

The important aspect of this scientific debate was that various researchers disagreed both in the nature of the action (cf. What was the bird doing?) and mental interpretation of the behaviour (cf. What kind of mental skill were involved?) shown in a short video. Importantly, such debates among fellow scientists are not particularly fruitful because in the absence of further data no resolution should be expected, and all parties are skilful to find supporting evidence for their case^16^. Moreover, scientists also may fall into the trap of not clarifying whether they rely on inductive or deductive reasoning in these debates. Actually, the present case makes both types of argument very difficult because such single anecdotes provide a very week strating point for an inductive approach. Similarly, it is difficult to contend that, for example, tool use in some specific bird species would justify the emergence of this skills in puffins.

In the present study, we used this case to explore whether the citizen science method may offer a solution^17^ for reaching a decision. Although this method has not gained large popularity in animal behaviour science, recently Root-Gutteridge et al.^18^ found it as a useful tool to judge some specific parameters of behaviour which are difficult to quantify by traditional methods.

However, rather than simply relying on the indiscriminate input of a wide audience, in this study we focused on the effect of anthropomorphism and professional experience. The action of the bird on the video published by Fayet et al.^11^ can be interpreted on the basis of three main aspects. First, the observer may prefer to remain at the level of the observable action (e.g. the puffin moves his head with a stick in its beak), as often suggested by ethologists^19^. Second, one may suggest a functional explanation (*teleological stance*^20^) on the goal of the puffin’s action (e.g. grooming or nest building). Third, the action may be described in terms of intentions to achieve a goal (*intentional stance*^21^; e.g. tool use). The preference for relying on teleological and intentional explanations is important feature of anthropomorphism^22,23^.

When facing a novel or particularly difficult problem, people often rely on the input of experienced persons, referred to as experts. The utilisation of expert opinion seems to be a natural choice for making educated decisions, because they are highly trained in a particular area of skill or knowledge, and are able to rely on much past experience compared to non-experts, that is, novices or lay people. Although research comparing experts and lay people has found some contradictory results that may depend on the specific task^24^, it is generally assumed that experts differ from novices in several ways^25^. For example, more experience makes experts organise their knowledge better, thus they detect relevant patterns earlier/faster than novices who are more focused on the actual perceivable cues^26^ and show better agreement^27^ compared to lay people.

In this study we aimed to find out whether two groups of experts (ethologists and ornithologists) and lay people differ in their account of the puffin anecdote (see above). Ethologists (people who have studied animal behaviour at university) are educated to refrain from (or to be sceptical about) functional and intentional explanation of behaviour in the absence of strong (experimental) evidence. Similar preference may be assumed for ornithologists who have a very broad experience with bird behaviour gained by observing a wide range of species for hundreds or thousands of hours. Thus both types of expertise facilitate a behavioural interpretation rather than relying on an anthropomorphic interpretation (see above). In contrast, lay people would be more prone to anthropomorphism and thus more inclined to interpret the puffin’s behaviour in terms of intentions.

In this study we provided all participants (who were unaware of the main goal of our data collection) a questionnaire that inquired about the (1) form of action (scratching/preening or nest-building), (2) the possible goal directedness (the stick was deployed to perform a goal-directed action) and the (3) certainty of the respondents’ opinion (for details see Methods).

We hypothesised that compared to unexperienced respondents (lay people), both groups of experts are more inclined to report nest-building behaviour rather than some form of grooming because the observed action on the video appears to be very different from scratching or preening behaviour in this species (see^10^), and using a stick for scratching has not been seen before in puffins (so the probability for such an action is tiny). In line with this, experts are expected to avoid reference to intention of the action because it could have been a lucky coincidence of events. Finally, based on their academic training experts should be more certain in their final judgement of the observed event. Thus, we expect a response with corresponding content from them to questions that ask about the causality of the behaviour (opinion about goal-directedness and absence of causal relationship). In contrast, lay observers may more frequently interpret the video recording as representing a goad-directed, intentional event of scratching.

## Methods

### Subjects

A total of 408 participants completed one of the two versions of the online questionnaire (*n* = 208 and 200 participants; Tables S1 and S2) between May and November of 2020. These respondents had different levels of expertise in ornithology and/or ethology.

The links of the questionnaire were advertised on different Hungarian ethological and ornithological mailing lists, and social media groups, and also students of BSc and MSc programs of ethology, biology, and ecology were involved in the survey (for details see Table S1).

### Questionnaire survey

We designed a Behaviour Evaluation Questionnaire (BEQ) with two variants that differed only in the attached video compilation. Both videos (Movies S1 and S2) had the following sequence:

1. Scratching (with text description and 10-s video of a scratching puffin)
2. Preening (with text description and 10-s video of a preening puffin)
3. The 11-s video published by Fayet et al.^11^ was repeated twice.

The scratching and preening parts of the compilations served as a reference of two typical behaviours which respondents could have relied on when evaluating the action seen on the third, Fayet et al.^11^ video. For both of the compilations, we used different scratching and preening videos (downloaded from YouTube) to avoid any bias.

At the beginning of the questionnaire, participants were asked to watch carefully the video compilation and then answer the questions. The videos were available to participants throughout the completion of the questionnaire. The questionnaires contained 15 items using a 5-point Likert scale (1 = strongly disagree, 2 = disagree, 3 = undecided, 4 = agree, 5 = strongly agree). These questions had to be answered in a fixed order right after viewing twice the video published by Fayet et al.^11^. The questionnaire consisted of three consecutive pages, and the respondents were able to go to the next pages when all questions were answered on the active page. The main purpose of this structure was to ensure that subsequent questions (especially the Q18 that mentions the term ‘tool use’) do not influence the respondents’ answers.

The questions targeted three main aspects (Table S2):

(i) *Goal-directedness:* grooming (e.g. the puffin is preening or scratching) versus nest-building;
(ii) *Intentionality:* was the action purposeful or accidental
(iii) *Neutral questions*: not related to the main focus of the questionnaire (e.g. Is the bird cute?).

In addition, apart from demographic questions (age, gender), we asked the respondents whether they had already read about tool use of puffins (in media or scientific publication), what level of experience they have in the fields of ethology, and ornithology. In the field of ethology respondents were asked to classify themselves into three categories: (i) no experience in ethology; (ii) interested in ethology and (iii) professional in ethology, while in the field of ornithology they could classify themselves into four groups: (i) no experience with birds, (ii) hobbyist birdwatcher, (iii) bird keeper and (iv) professional in ornithology.

### Statistical analyses

All the statistical analyses were evaluated by *R* statistical software^28^. First, we reversed the values of the answers belonging to the negative-wording question Q13 (e.g. we recoded values 5 to 1, 4 to 2 etc., henceforth ‘Q13rev’) to simplify the analyzes and make the results easier to interpret. To aid interpretation we also reworded this question at the presentation of our results. In the analyses, we took into account only the responders who have never read about the results of Fayet et al.^11^ or seen their video elsewhere (all who answered “no” for the questions *Q17* and *Q18*; *n* = 352 respondents). Then, we decided to reduce the number of variables by the means of a factor analysis in order to simplify our dataset to fewer and easier-to-interpret factors. We used Kaisler-Meyer-Olkin test (*KMO* score; *psych R package*;^29^) to determine the adequacy of the sample for further factor analyses. After the data reduction based on *h*^2^ values (items with *h*^2^ < 0.25 were removed) our data were adequate (*KMO* = 0.76) to perform the factor analysis. In these analyses we applied Varimax rotation and we obtained three factors (*likelihood chi*^2^ = 14.72, *p value* < 0.04; Table 1).

**Table 1:** The factor structure of the behaviour evaluation questionnaire (BEQ) after varimax rotation.

ID of the question	Questions (How certain you are that on the Video 3…)	Grooming (24.5%)	Nest building (20.9%)	Purposefulness (12.2%)
Q4	The bird builds a nest	− 0.22	**0.83**	− 0.10
Q5	The bird takes the stick into its beak to build a nest	− 0.23	**0.90**	− 0.15
Q6	The bird is preening	**0.57**	− 0.19	0.15
Q8	The bird takes the stick into its beak to scratch its feathers	**0.62**	− 0.19	0.34
Q10	The bird scratches its feathers with the stick	**0.75**	− 0.15	0.04
Q12	The bird accidentally scratches itself because the stick is right in its beak	− 0.10	0.25	− **0.51**
Q13rev*	The video reveals the purpose for which the bird took the stick into its beak	0.18	0.02	**0.72**
Q14	The bird is scratching	**0.74**	− 0.12	0.15

The labels of the factors were chosen based on the loading of items, and the proportion of the total variance explained by these factors are shown in parentheses. Loadings greater than 0.30 are highlighted in bold.

*Note that the Q13rev was originally a negative-wording question that we reversed the values of the answers before performing the statistical analyses. The original statement was: “The video does not reveal the purpose for which the bird took the stick into its beak”*.*

In the next step, we calculated Thurston’s or regression factor scores for each factor. This method is a complex algorithm that calculates factor scores by a linear combination of observed data, factor loads and the inverse of correlation matrix^30^. The inarguable advantage of this method (compared to e.g. Bartlett or Anderson–Rubin methods) is that is exact and takes into account the observed variables and factors separately and the correlations between them as well. Furthermore, this method is capable of maximizing the relationship between factor scores and factors (*maximal validity*). Although, it also has disadvantages (e.g. the estimated scores are not unbiased), it is a commonly used and recommended method to calculate factor scores for regression models^30,31^. Therefore, we decided to use this method for this purpose as well.

We used these scores as response variables in separate multiple regression models. Explanatory variables of each regression model were the experiences in ornithology (*Q19*; four-level factor: no experience, hobbyist birdwatcher, bird keeper and professional) and ethology (*Q20*; three-level factor: no experience, interested in and professional), the gender (*Q21*; two-level factor: male and female) and the age category (*Q22*; five-level factor: 18–24, 25–34, 35–44, 45–54, and 55 <) of the respondents, and we also included the version of the questionnaire (*Q23*; two-level factor: 1 and 2) in the models to control for the effects of any potential biases caused by the video compilation attached to the questionnaires.

The distributions of *grooming* and the *nest building* factor scores were skewed comparing with the normal distribution (skewness of grooming: − 0.55, *p value* < 0.001 and skewness of nest building: 0.70, *p value* < 0.001^32^). Therefore, for each factor, we have applied quantile regression (*quantreg R packages*^33^ in which the quantiles of the distribution of a factor score were estimated together with *pseudo R*^2^ values that are similar to *R*^2^ in simple linear regression models^34,35^.

In a further analysis, we selected three pairs of questions to examine the reliability of respondents belonging to different experience groups. To do this, we formed three pairs of questions in which the questions focused on the same topic: on scratching (*Q10* and *Q14*), the intentionality of the bird (e.g. the bird accidentally or intentionally picked up the stick and scratched itself; *Q8* and *Q12*), and the certainty of the respondent (e.g. how confident is the respondent that the video reveals what the bird is doing; *Q8* and *Q13rev*). Our goal was to detect the similarity of the answers respondents give to similar questions, thus testing the reliability of the respondents and the confidence of their answers (internal reliability). For example, if we get high values in reliability analyses, it means that the respondents gave very similar answers to similar questions, so we can assume that they are confident in their answers. To investigate their reliability we measured Cohen’s kappa values for each pairs of questions within different groups of experience: in the whole sample, as well as in specific groups like as lay (respondents who answered “no experience” to *Q19* and/or *Q20*) and expert people (who answered “professional” to *Q19* and/or *Q20*) both in ethology and ornithology (Table 2).

**Table 2:** The results of the Cohen’s kappa reliability analyses for the three pairs of questions within different groups of experience (*all*—all respondents, *expert—*“professional” answer to *Q19* and/or *Q20*; *lay—*“no experience” answer to *Q19* and/or *Q20*).

Groups	Scratching (Q10 and Q14)	Intentionality of the bird (Q8 and Q12)	Certainty of the respondent (Q8 and Q13rev)
All (N = 352)	**0.51**	− **0.27**	**0.33**
Experts in ornithology (N = 55)	**0.65**	− 0.35	**0.32**
Lays in ornithology (N = 118)	**0.47**	− 0.11	**0.33**
Experts in ethology (N = 39)	**0.66**	− 0.34	0.06
Lays in ethology (N = 90)	**0.48**	− 0.12	**0.19**
Experts both in ornithology and ethology (N = 20)	**0.56**	− 0.22	0.38
Lays both in ornithology and ethology (N = 60)	**0.41**	0.09	0.23

Statistically significant values (*p* < 0.05) are highlighted in bold.

In addition, using the bootstrap method, we compared the Cohen’s kappa values of lay and expert groups in the following steps: first, we randomly chose bootstrap samples from each group, and we calculated Cohen’s kappa values for each bootstrap sample separately. Secondly, we calculated the difference in Cohen’s kappa values between the lay and expert groups (e.g. experts in ethology minus lays in ethology) to determine the basic bootstrap confidence interval for the difference of Cohen’s kappa values (see also^36^).

### Ethics approval and consent to participate

The study was carried out in accordance with the recommendations of the Institutional Review Board of Institute of Biology, Eötvös Loránd University, Budapest, Hungary. The protocol was reviewed and approved by the Institutional Review Board of Institute of Biology, Eötvös Loránd University, Budapest, Hungary. The study was carried out in accordance with the Declaration of Helsinki. The Participants took part in the study voluntarily and anonymously, and provided their consent by clicking on the respective button provided by our electronic questionnaire form (reference number of the ethical permission: 2020/49).

## Results

### Factor analysis

After the KMO test, we retained 8 questionnaire items and we obtained three factors during the factor analysis (Table 1). These factors were labelled based on the loadings of the items, as follows: (i) *grooming* (4 items), (ii) *nest building* (2 items), and (iii) *purposefulness of the bird* (*henceforth* ‘purposefulness’) (2 items).

### Quantile regression analysis

The results of quantile regressions showed no significant difference between lays (‘no experience’ level) and experts (‘professional’ level) within either the ornithologist or ethologist experience groups in any of the three response variables (grooming, nest building, purposefulness, Table S3–S5). Although in the ‘building’ regression model some quantiles of ‘bird keepers’ (Table S4) significantly differed from lay ornithologists (‘no experience’ level), this difference was not observed in any other models or between the other levels of the two expertise groups (Tables S3–S5). Similarly, we did not find any differences between genders or age groups, nor did the version of the questionnaire have a significant effect on the analysed explanatory variables. Finally, we would note that all *pseudo R*^2^ values are very low in all models, suggesting that the explanatory power of the models is very low even with the most relevant variables available.

### Reliability measurements with Cohen’s Kappa coefficient

The results of Cohen’s kappa value measurements show that the reliability was the highest in the “scratching*”* question pair (Table 2). Here the Cohen’s kappa value was statistically significant in all of the examined expertise groups, where the reliability was the highest (substantial reliability based on^37^) within the two separate expert groups and it was moderate in all the other groups. However, the reliability of the respondents was lower in the *certainty of the respondent* question pair: the reliability of the different expertise groups ranged between poor and fair and we found statistically significant values only in some of the studied groups (see Table 2). Finally, we observed the lowest reliability in the question pair focusing on the *intentionality* of the bird, where almost all groups showed negative Cohen’s kappa values, indicating opposite answers to the questions, and only in the case of the whole sample we did find a statistically significant, but still negative reliability.

When we compared the reliability of the experiential groups for different pairs of questions, our results showed that there was no difference between lay and experienced respondents within either the ethological or the ornithological groups. We did not find any differences even when compared only those respondents who indicated themselves as experts or laymen in both fields of expertise (Table S6).

We observed a similar pattern in the responses to questions *Q1* and *Q2*, in which respondents were asked directly to compare the preening (*Q1*) and scratching (*Q2*) behaviour of puffins to the video published by Fayet et al. (2020). The distribution of their answers suggests that both lay people (ornithologists: *median* = 3, Interquartile range *‘IQR’* = 2; ethologists: *median* = 3, *IQR* = 2) and experts (ornithologists: *median* = 2, *IQR* = 2.25; ethologists: *median* = 3, *IQR* = 2) are uncertain that the target video resembles to the preening behaviour of puffins (*Q1*). We observed a similar pattern in both lays (ornithologists: *median* = 3, *IQR* = 2; ethologists: *median* = 3, *IQR* = 2) and experts (ornithologists: *median* = 2.5, *IQR* = 2.25; ethologists: *median* = 2, *IQR* = 2) when we asked them to compare the scratching behaviour of puffins to the target video (*Q2*).

## Discussion

In the present study, we used a citizen science approach to examine whether this method offers a more objective interpretation of the puffin anecdote published by Fayet et al.^11^. The two groups of experts and lay people did not differ in reporting that the bird was either grooming itself with the stick (scratching or preening) or performing a nest building behaviour. All groups showed similar level of uncertainty when asked about the goal-directedness of the displayed action (purposefulness). These findings were also supported by the subsequent reliability analyses. Both experts and lays were relatively confident in describing the behaviour as scratching. But this confidence was not observed when they had to make a direct comparison between a typical scratching or preening action (using the beak; *Q1* and *Q2*) and the target video (using a stick). Moreover, they provided contradictory answers to questions on goal-directedness (*Q8* and *Q12*) and having information about the causality of the action (*Q12* and *Q13rev*).

Overall, contrary to our expectation, the opinion of a large number of independent observers of the same event did not significantly contribute to the interpretation of the anecdote. Neither the potential proficiency of experts, nor the ‘openness’ of lay observers helped, so the original popularised assumption on tool use in puffins remained unsupported. However, despite of this negative outcome there is much to learn from these findings.

The capabilities of citizen science should be properly tested before professional utilisation. Lay people may be reliable when providing simple measures in numbers but their observational skills may be limited. Root-Gutteridge et al.^18^ found that naive citizen science observers could judge the intensity of dogs’ reaction to an auditory stimulus. They also revealed that the intensity of the judgment was positively associated with the number of different bodily reactions (e.g. breathing, eye/ear/body movement). So scientists may save some time of evaluating the dogs’ behaviour by this method but such data do not help to answer the mechanistic question on what the dog is actually doing when reacting to a sound stimulus or what kind of mental processes may operate. Thus, in some respect greater objectivity can be achieved by this method but only very specifically with regard to the question asked. Similarly, the lay people may agree in attributing the same simple or complex emotional states to animals^38^ but, again their concordance does not count as evidence for any underlying mental mechanism.

In general, some mild form of anthropomorphism may also play a role. Lay people express a preference to a shelter dog with a ball in its kennel, probably because people assume that it is a playful dog, even without having seen him playing^39^. Similar attributions may explain our results in this study because the fact that the bird had a stick in its beak made some people believe that it was used to scratching while others assumed that it was part of a nest-building action. Importantly, the timing, the shape and the way of execution of the action on the target video was very different from the typical scratching or preening actions typical for puffins (for the details see also^10^, one of which all observer had seen at the beginning of the questionnaire. Respondents were quite uncertain when we asked about the “similarity” (*Q1* and *Q2*) between the typical preening/scratching and the target video, probably because they could not choose between “similarity” in terms of body part movements and “similarity” in terms of function.

The lack of difference between experts and lay people led us to conclude that our experts were not experts in the strict sense of the definition^24^. We would note that by dividing the experience groups into a much finer scale, or even by testing the respondents’ experiences, we could have found the most qualified experts, then some differences could have emerged between these groups. With the emergence of artificial expert systems (e.g.^40^), it becomes more important to provide not only a definition for expertise but also to define the actual conditions under which an expert human or an artificial expert system can make reliable judgements and improve the decision making process. In short, three very important criteria have been put forward in this regard^24^.

First, the expert should be able to rely on accurate, relevant and objective data. Although, our experts had probably accidentally observed many instances of grooming (in birds and other animals) this experience was not systematically processed by them mentally. That is, they could not rely on an “annotated database” for making an objective judgement. This means that we should have involved experts with a massive training on puffin grooming behaviour (for a detailed ethological analysis of puffin grooming, see^10^).

Second, experts need to provide their judgements in a coherent and quantifiable way that can be eventually verified. Unfortunately, questionnaires of this type, which are typical in behavioural research^41^ do not meet this condition. Although, one could ask, for example, how many times the subjects raise their wing but concepts like “intentions” are not quantifiable this way: the action has either a goal or not.

Third, experts need to be able to rely on meaningful feedback about their judgements. Again, this was not possible in our case because experts had no previous possibility to find out whether “stick in the beak” consists a case for grooming. More importantly, with regard to cognitive aspects of animal behaviour it is close to impossible to get feedback helping to reveal the underlying mental control. Adult humans could provide an exception because they could be asked about the goals of their actions in retrospect.

The failure to meet the above (minimal) conditions explains why experts behave just like lay people despite their previous training, education and specific knowledge. These insights about experts strongly suggest that similar types of publications that are associated with experienced scientists do not make them more reliable in terms of judgement, and, in addition, a small number, closely associated expert researchers may be unaware of forming the same opinion easier and finding a familiar pattern in random noise^42^. Accordingly, such observations may have a place in some data repositories but publications should be avoided or preceded by a thorough and critical analysis, and presented as a hypothesis^10^.

In summary, we seem to have to face the fact that in animal/human cognition there are no experts in strict sense when it comes to explaining mental phenomena based on single or even multiple anecdotes. Experts are not better than lays and all explanations rely on subjective (biased) opinions.

## Conclusions

In line with the previous, more subjective evaluation^10^, these results strongly emphasise that anecdotes lack the power for being analysed in any deeper way apart from providing a description of the action and the context.

In our view determining the certainty of the subjects’ answers in citizen science projects could be a critical part of their usefulness. This can be done relatively easily by using specific items in the questionnaire that relate to the same target variable. Judgements of observers should be only relied upon if their confidence exceeds a specific limit^37^.

Furthermore, the role of experts in the behavioural sciences should also be investigated in more detail. One could test the capacity of experts to improve in comparison to lay people. These insights may also help in constructing artificial expert systems that may ease the burden of behavioural analysis. But neither type of expert will be able to directly reflect on mental mechanisms of the behaviour under study.

## Supplementary Information

Supplementary Information.
