# Author Correction: Uncertainty in experts’ judgments exposes the vulnerability of research reporting anecdotes on animals’ cognitive abilities

**DOI:** 10.1038/s41598-021-97806-2

**Published:** 2021-09-03

**Authors:** Krisztina Sándor, Balázs Könnyű, Ádám Miklósi

**Affiliations:** 1 Behavioural Ecology Research Group, Center for Natural Sciences, University of Pannonia, Veszprém, Hungary; 2 MTA-ELTE Comparative Ethology Research Group, Budapest, Hungary; 3 Department of Plant Systematics, Ecology and Theoretical Biology, Eötvös Loránd University, Budapest, Hungary; 4 Institute of Evolution, Eötvös Loránd Research Network, Budapest, Hungary; 5 Department of Ethology, Eötvös Loránd University, Budapest, Hungary

Correction to: *Scientific Reports* 10.1038/s41598-021-95384-x, published online 10 August 2021

The original version of this Article contained an error in Reference 9, which was incorrectly given as:

Sarringhaus, L. A., Stock, J. T., Marchant, L. F. & McGrew, W. C. Bilateral asymmetry in the limb bones of the chimpanzee (*Pan troglodytes*). *Am. J. Phys. Anthropol.* **128**, 840–845 (2005).

The correct reference is listed below:

Sarringhaus, L. A., McGrew, W. C., & Marchant, L. F. Misuse of anecdotes in primatology: lessons from citation analysis. *Am. J.Primatol.* **65**(3), 283–288 (2005).

The original Article has been corrected.

