# Supplementary material for: Uncertainty in experts’ judgments exposes the vulnerability of research reporting anecdotes on animals’ cognitive abilities
Source: Sci Rep. 2021 Aug 10;11:16255. doi: 10.1038/s41598-021-95384-x (PMC8355131; doi:10.1038/s41598-021-95384-x)
Supplement: Supplementary file 1 — Supplementary Information. [file 41598_2021_95384_MOESM1_ESM.docx]

**APPENDIX**

**Uncertainty in expert’s judgments exposes the vulnerability of research reporting anecdotes on animals’ cognitive abilities**

KRISZTINA SÁNDOR, BALÁZS KÖNNYŰ, ÁDÁM MIKLÓSI

**Table S1.** Sample characteristics of the questionnaire. During the statistical analyses we took into account only those respondents who have never read about the results of Fayet et al. (2020) or seen their video elsewhere. Thus, we analysed the responses of 352 participants out of the initial 408 respondents.

| **Experience in ornithology; N (%)** | | |
| --- | --- | --- |
|  | No experience | **118 (33.5%)** |
|  | Hobbyist | **140 (39.8%)** |
|  | Keeper | **39 (11.1%)** |
|  | Professional | **55 (15.6%)** |
| **Experience in ethology; N (%)** | | |
|  | No experience | **90 (25.6%)** |
|  | Interested | **223 (63.4%)** |
|  | Professional | **39 (11.1%)** |
| **Gender; N (%)** | |  |
|  | Male | **137 (38.9%)** |
| **Age categories; N (%)** | |  |
|  | 18-24 | **93 (26.4%)** |
|  | 25-34 | **96 (27.3%)** |
|  | 35-44 | **74 (21%)** |
|  | 45-54 | **49 (13.9%)** |
|  | 55- | **40 (11.4%)** |

**Table S2.** The items of the original BEQ and the IDs associated to them. After Kaisler-Meyer-Olkin analyses we retained 8 questionnaire items (highlighted in bold) corresponding to three factors: i) *grooming* (“groom”), ii) *nest buildin*g (“nest”) and iii) purposefulness of the bird (“purpose”)

| **ID of the question** | **Corresp. factor** |  | **Questions** |
| --- | --- | --- | --- |
|  |  | How certain you are that on the Video 3:  *(five-level items: strongly disagree – disagree – undecided – agree – strongly agree)* | |
| **Q1** |  |  | The behaviour of the bird is similar to that seen on Video 1. |
| **Q2** |  |  | The behaviour of the bird is similar to that seen on Video 2. |
| **Q3** |  |  | The bird takes a stick into its beak |
| **Q4** | **Nest** |  | **The bird builds a nest** |
| **Q5** | **Nest** |  | **The bird takes the stick into its beak to build a nest** |
| **Q6** | **Groom** |  | **The bird is preening** |
| **Q7** |  |  | The bird accidentally takes the stick into its beak |
| **Q8** | **Groom** |  | **The bird takes the stick into its beak to scratch its feathers** |
| **Q9** |  |  | The bird toddles with the stick in its beak |
| **Q10** | **Groom** |  | **The bird scratches its feathers with the stick** |
| **Q11** |  |  | The bird is very cute |
| **Q12** | **Purpose** |  | **The bird accidentally scratches itself because the stick is right in its beak** |
| **Q13*** | **Purpose** |  | **The video does not reveal the purpose for which the bird took the stick into its beak** |
| **Q14** | **Groom** |  | **The bird is scratching** |
| **Q15** |  |  | The bird is just playing with the stick |
| **Q16** |  | Have you ever seen this bird species (live or in a movie)? *(yes / no)* | |
| **Q17** |  | Have you ever seen Video 3? *(yes / no)* | |
| **Q18** |  | Have you read about the tool use of puffins (media, scientific publication)? *(yes / no)* | |
| **Q19** |  | How experienced are you in ornithology? *(no exp. / hobbyist / bird keeper / professional)* | |
| **Q20** |  | How experienced are you in ethology? *(no exp. / interested / professional)* | |
| **Q21** |  | What is your gender? *(male / female)* | |
| **Q22** |  | How old are you? *(18-24 / 25-34 / 35-44 / 45-54/ 55- )* | |

***** Note that as the Q13 is a negative-wording question, we reversed the values of the answers of it before performing the statistical analyses.

**Movie S1.** The video compilation attached to the first version of the questionnaire (BEQ1)

<https://youtu.be/wXM64OmXzbQ>

**Movie S2.** The video compilation attached to the first version of the questionnaire (BEQ2)

<https://youtu.be/JNcldo5GctI>

**Table S3.** Results of quantile regression of the *grooming* factor. Statistically significant coefficients are highlighted in bold. In the intercept: Experience in ornithology (*Q19*) “*no experience”*; Experience in ethology (*Q20*) “*no experience”*; Gender (*Q21*) “*female”*; Age (*Q22*) “*18-24”*; Version of the BEQ (*Q23*) “*1”*.

| **Explanatory variables** | | **Quantiles** | | | | |
| --- | --- | --- | --- | --- | --- | --- |
|  |  | **0.1** | **0.25** | **0.5** | **0.75** | **0.9** |
| **Intercept** | | -0.53 | -0.12 | **0.6** | **0.89** | **1.23** |
| **Experience in ornithology (*Q19*)** | |  |  |  |  |  |
|  | Hobbyist | -0.12 | -0.24 | 0.09 | 0.15 | -0.02 |
|  | Bird keeper | -0.02 | -0.06 | -0.18 | 0.03 | -0.05 |
|  | Professional | -0.32 | -0.5 | 0.14 | 0.25 | -0.2 |
| **Experience in ethology (*Q20*)** | |  |  |  |  |  |
|  | Interested | -0.12 | 0.04 | -0.18 | 0.03 | -0.05 |
|  | Professional | -0.43 | 0.03 | -0.24 | -0.17 | -0.1 |
| **Gender (*Q21*)** | |  |  |  |  |  |
|  | Male | 0.1 | -0.05 | -0.14 | **-0.27** | -0.07 |
| **Age (*Q22*)** | |  |  |  |  |  |
|  | 25-34 | -0.26 | -0.23 | -0.21 | -0.13 | -0.03 |
|  | 35-44 | -0.61 | -0.11 | -0.28 | -0.1 | 0.03 |
|  | 45-54 | **-0.83** | **-0.64** | **-0.62** | **-0.58** | -0.09 |
|  | 22 >55 | -0.42 | -0.23 | -0.42 | -0.26 | -0.06 |
| **Version of the BEQ (*Q23*)** | |  |  |  |  |  |
|  | 2 | -0.15 | -0.16 | -0.18 | -0.04 | -0.09 |
| **Pseudo *R^2^*** | | 0.06 | 0.01 | 0.04 | 0.08 | 0.01 |

**Table S4.** Results of quantile regression of the *nest* *building* factor. Statistically significant coefficients are highlighted in bold. In the intercept: Experience in ornithology (*Q19*) “*no experience”*; Experience in ethology (*Q20*) “*no experience”*; Gender (*Q21*) “*female”*; Age (*Q22*) “*18-24”*; Version of the BEQ (*Q23*) “*1”*.

| **Explanatory variables** | | **Quantiles** | | | | |
| --- | --- | --- | --- | --- | --- | --- |
|  |  | **0.1** | **0.25** | **0.5** | **0.75** | **0.9** |
| **Intercept** | | **-0.95** | **-0.36** | 0.18 | **0.65** | **1.46** |
| **Experience in ornithology (*Q19*)** | |  |  |  |  |  |
|  | Hobbyist | 0.05 | 0.07 | -0.02 | 0.23 | 0.14 |
|  | Bird keeper | 0.05 | 0.07 | **0.6** | **0.53** | **0.53** |
|  | Professional | 0.16 | 0.1 | -0.23 | 0.18 | 0.28 |
| **Experience in ethology (*Q20*)** | |  |  |  |  |  |
|  | Interested | 0.07 | -0.09 | -0.23 | -0.25 | -0.39 |
|  | Professional | -0.25 | **-0.4** | -0.27 | -0.11 | -0.08 |
| **Gender (*Q21*)** | |  |  |  |  |  |
|  | Male | -0.02 | -0.04 | 0.04 | 0.13 | -0.13 |
| **Age (*Q22*)** | |  |  |  |  |  |
|  | 25-34 | -0.2 | -0.15 | -0.02 | -0.12 | -0.15 |
|  | 35-44 | -0.19 | -0.26 | 0 | 0.05 | -0.26 |
|  | 45-54 | -0.34 | **-0.35** | -0.34 | -0.29 | 0.35 |
|  | 22 >55 | -0.07 | 0.1 | 0.11 | 0.21 | **1.15** |
| **Version of the BEQ (*Q23*)** | |  |  |  |  |  |
|  | 2 | -0.08/ | **-0.31** | -0.28 | -0.08 | -0.24 |
| **Pseudo *R^2^*** | | 0.02 | 0.02 | 0.04 | 0.01 | 0.03 |

**Table S5.** Results of quantile regression of the *purposefulness of the bird* factor. Statistically significant coefficients are highlighted in bold. In the intercept: Experience in ornithology (*Q19*) “*no experience”*; Experience in ethology (*Q20*) “*no experience”*; Gender (*Q21*) “*female”*; Age (*Q22*) “*18-24”*; Version of the BEQ (*Q23*) “*1”*.

| **Explanatory variables** | | **Quantiles** | | | | |
| --- | --- | --- | --- | --- | --- | --- |
|  |  | **0.1** | **0.25** | **0.5** | **0.75** | **0.9** |
| **Intercept** | | **-0.93** | **-0.65** | -0.23 | **0.57** | **1.08** |
| **Experience in ornithology (*Q19*)** | |  |  |  |  |  |
|  | Hobbyist | -0.1 | -0.02 | -0.09 | -0.04 | -0.06 |
|  | Bird keeper | -0.14 | -0.18 | -0.22 | -0.16 | **-0.41** |
|  | Professional | 0.02 | 0.05 | -0.04 | -0.23 | -0.3 |
| **Experience in ethology (*Q20*)** | |  |  |  |  |  |
|  | Interested | 0.09 | 0.03 | 0.29 | 0.33 | 0.18 |
|  | Professional | 0.1 | 0.19 | 0.3 | 0.34 | -0.16 |
| **Gender (*Q21*)** | |  |  |  |  |  |
|  | Male | -0.06 | 0.01 | -0.04 | -0.18 | 0.19 |
| **Age (*Q22*)** | |  |  |  |  |  |
|  | 25-34 | 0.09 | 0.07 | 0.15 | -0.24 | -0.19 |
|  | 35-44 | -0.03 | -0.2 | -0.37 | -0.33 | -0.08 |
|  | 45-54 | **-0.24** | 0.07 | -0.26 | 0.04 | 0.02 |
|  | 22 >55 | 0.25 | **0.46** | 0.32 | 0.12 | 0.21 |
| **Version of the BEQ (*Q23*)** | |  |  |  |  |  |
|  | 2 | -0.08 | 0.04 | 0.06 | 0.01 | 0.02 |
| **Pseudo *R^2^*** | | -0.02 | 0.07 | 0.05 | 0.04 | 0.01 |

**Table S6.** Results of comparisons of the reliability of lay and experienced respondents for the three pairs of questions (for more details see Methods). The basic bootstrap method was used to determine the confidence interval of the difference of Cohen’s kappa values.

|  | **Scratching**  **(Q10 and Q14)** | | | **Intentionality**  **(Q8 and Q12)** | | | **Certainty of the respondent**  **(Q8 and Q13rev)** | | |
| --- | --- | --- | --- | --- | --- | --- | --- | --- | --- |
|  | **Lower** | **Estimate** | **Upper** | **Lower** | **Estimate** | **Upper** | **Lower** | **Estimate** | **Upper** |
| **Ethologist** | -0.7 | 0.19 | 0.49 | -0.61 | -0.22 | 0.14 | -0.51 | -0.13 | 0.25 |
| **Ornithologist** | -0.05 | 0.19 | 0.44 | -0.55 | -0.24 | 0.04 | -0.31 | -0.01 | 0.32 |
| **Both** | -0.20 | 0.15 | 0.57 | -0.80 | -0.30 | 0.16 | -0.30 | 0.15 | 0.66 |
